# Increased C-reactive protein is associated with the severity of thoracic radiotherapy-induced cardiomyopathy

**DOI:** 10.1186/s40959-020-0058-1

**Published:** 2020-02-28

**Authors:** Justin M. Canada, Georgia K. Thomas, Cory R. Trankle, Salvatore Carbone, Hayley Billingsley, Benjamin W. Van Tassell, Ronald K. Evans, Ryan Garten, Elisabeth Weiss, Antonio Abbate

**Affiliations:** 1 VCU Pauley Heart Center, Virginia Commonwealth University, P.O. Box 980335, 1200 E. Broad Street, Richmond, Virginia, 23298 USA; 2 Department of Kinesiology & Health Sciences, College of Humanities & Sciences, Virginia Commonwealth University, Richmond, Virginia, USA; 3 Department of Pharmacotherapy and Outcome Sciences, Virginia Commonwealth University, Richmond, Virginia, USA; 4 Department of Radiation Oncology, Virginia Commonwealth University, Richmond, Virginia, USA; 5 C. Kenneth and Dianne Wright Center for Clinical and Translational Research, Virginia Commonwealth University, Richmond, Virginia, USA

**Keywords:** Radiotherapy, Cardiotoxicity, Inflammation

## Abstract

**Background:**

Irradiation of the heart during cancer radiotherapy is associated with a dose-dependent risk of heart failure. Animal studies have demonstrated that irradiation leads to an inflammatory response within the heart as well as a reduction in cardiac reserve. In the current study we aimed to evaluate whether inflammatory biomarkers correlated with changes in cardiac function and reserve after radiotherapy for breast or lung cancer.

**Methods and results:**

We studied 25 subjects with a history of breast or lung cancer without a prior diagnosis of cardiovascular disease or heart failure, 1.8 years [0.4–3.6] post-radiotherapy involving at least 5 Gray (Gy) to at least 10% of the heart. High-sensitivity C-reactive protein (CRP) was abnormal (≥2 mg/L) in 16 (64%) subjects. Cardiac function and reserve was measured with Doppler echocardiography before and after exercise and defined as left-ventricular ejection fraction (LVEF), early diastolic mitral annulus velocity (e’), and increase in LV outflow tract velocity time integral cardiac output (cardiac reserve) with exercise. Subjects with abnormal CRP had significantly lower LVEF (51 [44–59] % vs 61 [52–64] %, *P* = 0.039), lower e’ (7.4 [6.6–7.9] cm/sec vs 9.9 [8.3–12.0] cm/sec, *P* = 0.010), and smaller cardiac reserve (+ 1.5 [1.2–1.7] L/min vs + 1.9 [1.7–2.2] L/min, *P* = 0.024).

**Conclusion:**

Elevated systemic inflammation is associated with impaired left-ventricular systolic and diastolic function both at rest and during exercise in subjects who have received radiotherapy with significant incidental heart dose for the treatment of cancer.

## Introduction

Radiotherapy remains a cornerstone of treatment for many cancers. Lung, esophageal, breast, and proximal gastric cancers still receive incidental radiation to the heart as part of curative intent or palliative care [1–4]. Contemporary and more sophisticated administration of thoracic radiotherapy and systemic immunotherapy have been effective in reducing cancer-related mortality and limiting exposure to the heart. However, radiotherapy to the chest increases the risk for cancer-unrelated morbidity and mortality, especially cardiovascular mortality, in a dose-dependent manner [1, 5–8]. Furthermore, recent studies have shown that major adverse cardiovascular events, like acute myocardial infarction and stroke, are likely occurring earlier post-treatment than previously thought [1, 5]. In a well-characterized cohort of 945 women with breast cancer who had received a mean radiation dose to the heart of 2.5 Gray (Gy), Saiki et al. showed that 60 patients (6%) developed new-onset heart failure (HF) 5.8 ± 3.4 years following radiotherapy [9]. When a nested case-control matched analysis was performed, the mean heart dose was higher in HF cases (3.3 ± 2.7 Gy) than controls (2.1 ± 2.0 Gy; *P* = 0.004), and the odds ratio (95% confidence interval) for HF per log-change in mean cardiac radiation dose was 9.1 (3.4–24.4), thus highlighting that even relatively small doses of radiotherapy to the heart have the potential to cause HF [9]. Moreover, exercise intolerance, a surrogate of impaired cardiac reserve and a strong predictor of all-cause and cancer-related mortality [10, 11], seems to appear early after radiotherapy and may contribute significantly to impairments in quality of life [12–15].

Commonly used tools to assess cardiac function (i.e., resting left-ventricular ejection fraction [LVEF] by echocardiography) are notoriously insensitive to minor injury, and therefore subtle changes can go unnoticed. Moreover, a large portion of patients presenting with new-onset heart failure are expected to have preserved LVEF [16]. In Saiki et al., 64% of patients with new-onset HF after radiotherapy had preserved LVEF [9]. Using cardiopulmonary exercise testing, we have recently described an early dose-dependent inverse relationship between radiation dose to the heart and peak oxygen consumption in patients who had received radiotherapy to the chest driven primarily by impairments in cardiac diastolic reserve [13].

The mechanisms by which radiotherapy induces impaired cardiac reserve is complex. Preclinical studies have shown that there is an acute series of events following radiotherapy characterized by inflammation resulting in impaired contractile reserve, followed by cell death leading to a reparative fibrotic response in the pericardium, myocardium and valvular structures [17–20]. Activation of pro-inflammatory pathways likely play an important role in the early changes seen following radiotherapy. Animal models have demonstrated reversible systolic dysfunction and reduced LV contractility reserve following injections of interleukin (IL)-1**β**, the prototypical pro-inflammatory cytokine, in otherwise healthy mice, whereas mice pretreated with anakinra, an IL-1 receptor antagonist, or an IL-1**β** antibody, were spared from these detrimental effects [21]. Additionally, mice injected with plasma from patients with stable chronic systolic HF and elevated plasma levels of C-reactive protein (CRP) showed normal resting systolic function but significantly impaired contractile reserve [22]. In the current study we sought to determine whether CRP, a systemic inflammatory biomarker and surrogate for IL-1 activity, could identify patients with radiotherapy-induced impairment in cardiac function or reserve.

## Methods

We conducted a single-center prospective study enrolling patients with a history of breast or lung cancer who had received thoracic radiotherapy with a resultant significant cardiac dose (at least 5 Gy to at least 10% of the heart) as part of intended curative treatment for malignancy. These subjects did not have a prior diagnosis of cardiovascular disease or heart failure. All patients were at least 18 years of age, had adequate acoustic windows for echocardiography, and had to be able to perform treadmill exercise testing with ventilatory gas-analysis. All patients underwent informed consent prior to enrollment. The study was approved by the Virginia Commonwealth University Institutional Review Board.

A blood sample was obtained to evaluate the biomarker high-sensitivity CRP (hsCRP). Elevated systemic inflammation was defined as an hsCRP ≥2.0 mg/L [23]. All patients underwent transthoracic Doppler echocardiography at rest and immediately post-exercise to evaluate cardiac systolic and diastolic function. Symptom-limited exercise was performed utilizing a conservative treadmill ramping protocol using percentage of age-predicted maximal heart rate (%APMHR) to quantify subject effort. Tissue Doppler-derived early transmitral flow velocity (E), early diastolic mitral annular velocities (e′) averaged between the lateral and septal annulus – a measure of diastolic function - and the change in left-ventricular outflow tract velocity time integral cardiac output (∆ LVOT VTI CO) with exercise – a measure of cardiac reserve - were obtained according to standard recommendations [24, 25]. Since estimation of the cross-sectional area of the left-ventricular outflow tract represents a potential source of error, the velocity time integral alone was used as a surrogate for cardiac output measurement [26].

Data are reported as median and interquartile range [IQR] for potential deviation from a Gaussian distribution. Spearman correlation coefficients were estimated to assess correlations between CRP and cardiac function from echocardiography variables. A Mann-Whitney U test was performed to compare those with and without elevated systemic inflammation (hsCRP ≥2.0 mg/L versus < 2.0 mg/L). Fisher’s exact test was used to assess differences in the presence of categorical (Yes/ No) comorbid conditions based on medical history (prior chemotherapy, cancer type (breast vs. lung), anemia, obesity, hypertension, dyslipidemia, diabetes mellitus, current smoker, sedentary lifestyle) or agents that have a known modifying-association with CRP (i.e., statins, hormone therapy) between those with and without elevated hsCRP.

## Results

Table 1 provides the demographic and clinical characteristics of the entire cohort.

**Table 1:** Characteristics of the Cohort

Variables	Median [IQR] or N (%)
Age, years	63 [59–66]
Female, n (%)	15 (60%)
Caucasian, n (%)	16 (64%)
African-American, n (%)	9 (36%)
Body mass index, kg/m^2^	26.4 [22.6–30.2]
Cancer Type	
Lung	15 (60%)
Breast	10 (40%)
Time since Cancer Diagnosis, years	2.4 [1.1–3.9]
Prior chemotherapy	21 (84%)
Time since completion of chemotherapy, years	1.5 [0.5–3.0]
Time since completion of Radiotherapy, years	1.8 [0.4–3.6]
Hormonal modulating therapy (Breast cancer only)	7 (28%)
MCRD, Gy	5.4 [3.7–14.7]
C-reactive protein, mg/L	3.0 [1.7–6.9]
%APMHR	93 [78–102]
LVEF, %	52 [47–61]
Doppler echo e’ velocity (cm/sec)	7.6 [7.0–9.6]
Delta LVOT VTI CO (L/min)	1.6 [1.5–1.9]

Data are listed as n (%) or median (interquartile range). *Abbreviations*: *kg/m*^*2*^ Kilograms per meter squared, *MCRD* Mean cardiac radiation dose, *Gy* Gray units, *mg/L* Milligrams per liter, *%APMHR* Percentage of age-predicted maximal heart rate, *LVEF* Left-ventricular ejection fraction, *e*’ Doppler early diastolic mitral annular velocity, *cm/sec* Centimeters per second, *LVOT VTI CO* Left-ventricular outflow tract velocity time integral cardiac output with exercise, *L/min* Liters per minute.

Sixteen (64%) subjects had elevated hsCRP (≥2.0 mg/L). The %APMHR achieved during exercise testing was not different between those with and without elevated hsCRP (93 [81–101] % vs. 95 [74–110] %, *P* = 0.742). Additionally, there were no significant differences between those with and without elevated hsCRP respective to presence of comorbid conditions, statin, or hormone therapy use (Table 2). Finally, none of the subjects had known active or progressive disease at the time of study participation.

**Table 2:** Distribution of comorbid conditions or statin use in those without and with elevated hsCRP

Categorical Variables	hsCRP < 2 mg/L (*n* = 9)	hsCRP ≥ 2 mg/L (*n* = 16)	*P*-value
History of chemotherapy	7 (78%)	14 (88%)	0.602
Cancer type (Breast vs. Lung)			1.000
Breast cancer	4 (44%)	6 (38%)	
Lung cancer	5 (56%)	10 (63%)	
History of anemia	1 (11%)	4 (25%)	0.621
Obesity	1 (11%)	6 (38%)	0.355
Hypertension	4 (44%)	10 (63%)	0.434
Dyslipidemia	5 (56%)	5 (31%)	0.397
Diabetes Mellitus	1 (11%)	5 (31%)	0.364
Current Smoker	2 (22%)	4 (25%)	1.000
Sedentary Lifestyle	3 (33%)	8 (50%)	0.677
Statin Use	3 (33%)	5 (31%)	1.000
Hormone therapy	3 (33%)	4 (25%)	0.673

Legend: Distribution of nominal variables expressed as Yes or No based on medical history and/or medication use. Proportion of those with the presence of categorical comorbid conditions, statin use, or hormone therapy use are reported as number (%). Obesity defined as a body mass index ≥30 kg per meter squared

**Fig. 1:**
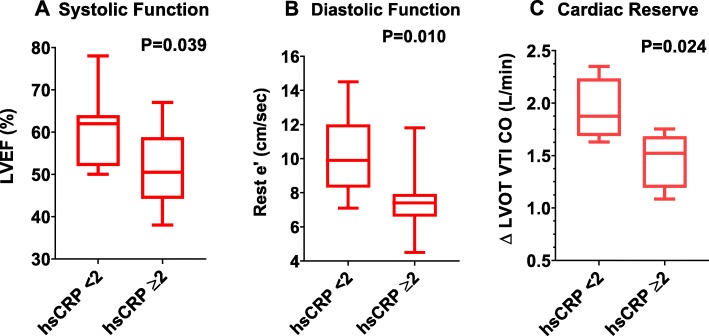
Impaired cardiac function in patients with elevated C-reactive protein levels after radiotherapy therapy for cancer. Transthoracic Doppler echocardiography at rest and at immediately post-exercise was used to measured left ventricular ejection fraction (Panel **a**), mitral annulus early diastolic velocity for myocardial relaxation (Panel **b**), and the change in left-ventricular outflow tract velocity time integral cardiac output (Panel **c**) with exercise as a measure of cardiac reserve. Subjects with elevated C-reactive protein (CRP) levels showed significantly worse impairments in cardiac systolic and diastolic function. Abbreviations: LVEF = left-ventricular ejection fraction; hsCRP = high-sensitivity C-reactive protein; e’ = early diastolic mitral annular velocities averaged between the lateral and septal annulus; ∆ LVOT VTI CO = delta left-ventricular outflow tract velocity time integral cardiac output with exercise

**Fig. 2:**
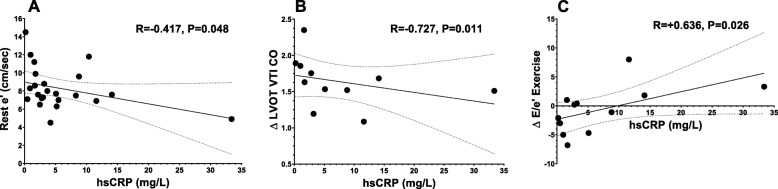
Correlations of cardiac function with high-sensitivity C-reactive protein. High-sensitivity CRP levels as a continuous variable inversely correlated with the Doppler e’ velocity (Panel **a**) and ∆ LVOT VTI CO with exercise (Panel **b**), and directly with the ∆ exercise E/e’ ratio (Panel **c**). Abbreviations: e’ = early diastolic mitral annular velocities averaged between the lateral and septal annulus; hsCRP = high-sensitivity C-reactive protein; ∆ LVOT VTI CO = delta left-ventricular outflow tract velocity time integral cardiac output with exercise; ∆ E/e’ exercise = delta early transmitral flow to early diastolic mitral annular velocity ratio with exercise

Patients with elevated hsCRP had significantly lower LVEF (51 [44–59] % vs. 61 [52–64] %, *P* = 0.039), e’ velocity (7.4 [6.6–7.9] cm/sec vs. 9.9 [8.3–12.0] cm/sec, *P* = 0.010), and ∆ LVOT VTI CO with exercise (+ 1.5 [1.2–1.7] L/min vs + 1.9 [1.7–2.2] L/min, *P* = 0.024), respectively compared to those with hsCRP < 2 mg/L. Figure 1 illustrates the differences in LVEF (**Panel A**), impaired diastolic function as shown by lower e’ values (**Panel B**), and reduced cardiac reserve (**Panel C**). In Fig. 2, hsCRP levels as a continuous variable inversely correlated with the Doppler e’ velocity (R = -0.417, *P* = 0.048; **Panel A**) and ∆ LVOT VTI CO with exercise (R = -0.727, *P* = 0.011; **Panel B**), and directly with the Doppler-derived estimated intra-cardiac pressures with exercise, ∆ exercise E/e’ ratio (R = + 0.636, *P* = 0.026; **Panel C**).

## Discussion

Radiotherapy is an integral treatment modality for many cancers. The relationship between ionizing radiation, inflammation and cardiotoxicity is complex and incompletely understood [27]. A number of acute effects including endothelial damage followed by inflammatory cell infiltration with subsequent fibrotic changes have been described. Systemic inflammation following radiotherapy has been associated with transient cardiac dysfunction including HF [17] and elevated pre-treatment serum CRP levels have been associated with poorer prognosis in esophageal cancer patients [28]. Moreover, cross-sectional studies in breast cancer survivors have shown a correlation between elevation in the pro-inflammatory markers CRP and IL-1 receptor antagonist and persistent post-treatment fatigue [29].

Here we show that elevated CRP levels are common and correlate with measures of impaired cardiac function in patients who have received thoracic radiotherapy for the treatment of cancer. These results suggest that changes in CRP and cardiac function can serve as early markers of cardiotoxicity following radiotherapy, and could potentially serve as useful biomarkers for outcome prediction. The limitations of this study are its small sample size and cross-sectional nature thus the observed associations do not prove causality.

In conclusion, further studies are needed to develop improved detection methods and ultimately treatments for subclinical cardiotoxicity in patients who have undergone radiation therapy to prevent long-term cardiac sequelae. Gaining a better understanding of the relationship between CRP and cardiac function in patients who have undergone radiation therapy could potentially help to optimize treatment, and ideally, serve as therapeutic targets to minimize long-term unwanted cardiac side-effects. IL-1 blockers are being studied for the prevention and treatment of heart failure, showing a promising safety and efficacy profile [30–35] thus introducing the possibility of future clinical trials investigating IL-1 blockade to treat patients at risk for radiation-induced heart failure.

## Data Availability

The dataset analyzed during the current study are available from the corresponding author upon reasonable request.

## Abbreviations

∆: Delta
E: Early transmitral velocity
e’: Early diastolic mitral annular velocity
Gy: Gray
HF: Heart failure
hsCRP: High-sensitivity C-reactive protein
IL-1: Interleukin-1
IQR: Interquartile range
LVEF: Left-ventricular ejection fraction
LVOT VTI CO: Left-ventricular outflow tract velocity time integral cardiac output
MCRD: Mean cardiac radiation dose
